# Supplementary material for: Distinct roles for innexin gap junctions and hemichannels in mechanosensation
Source: eLife. 2020 Jan 29;9:e50597. doi: 10.7554/eLife.50597 (PMC7010410; doi:10.7554/eLife.50597)
Supplement: Supplementary file 1. [file elife-50597-supp1.docx]

**Supplementary File 1. Strains used in this study.**

| **Strain** | **Genotype** |
| --- | --- |
| AQ906 (Suzuki et al., 2003) | bzIs17[*Pmec-4*::*yc2.12*, *lin-15*(+)] |
| AQ2993 | bzIs17[*Pmec-4*::*yc2.12*, *lin-15*(+)] ljEx498[*Pmec-7*::*unc-9* RNAi, *Punc-122*::*gfp*] |
| AQ2995 | bzIs17[*Pmec-4*::*yc2.12*, *lin-15*(+)] ljEx500[*Pmec-7*::*unc-7* RNAi, *Punc-122*::*gfp*] |
| AQ2998 | bzIs17[*Pmec-4*::*yc2.12*, *lin-15*(+)] ljEx503[*Pmec-7*::*inx-7* RNAi, *Punc-122*::*gfp*] |
| AQ2987 | bzIs17[*Pmec-4*::*yc2.12*, *lin-15*(+)] ljEx492[*Pmec-7*::*E.coli Cat1* RNAi, *Punc-122*::*gfp*] |
| CB101 (Brenner, 1974) | *unc-9(e101)* |
| AQ3249 | *unc-9(e101),* bzIs17[*Pmec-4*::*yc2.12*, *lin-15*(+)] |
| CB5 (Brenner, 1974) | *unc-7(e5)* |
| AQ3248 | *unc-7(e5)* bzIs17[*Pmec-4*::*yc2.12*, *lin-15*(+)] |
| AQ3241 | *unc-7(e5)* bzIs17[*Pmec-4*::*yc2.12*, *lin-15*(+)] ljEx611[*Pmec-4*::*unc-7a*:: SL2::*tag rfp*, *Punc-122*::*gfp*] |
| AQ3243 | *unc-7(e5)* bzIs17[*Pmec-4*::*yc2.12*, *lin-15*(+)] ljEx613[*Pmec-4*::*unc-7a* C173A, C191A, C377A, C394A:: SL2::*tag rfp*, *Punc-122*::*gfp*] |
| AQ3775 | *unc-7(e5)* bzIs17[*Pmec-4*::*yc2.12*, *lin-15*(+)] ljEx772[*Pmec-4*::*unc-7c*:: SL2::*tag rfp, Punc-122::mcherry*] |
| AQ3738 | *unc-7(e5)* bzIs17[*Pmec-4*::*yc2.12*, *lin-15*(+)] ljEx970[*Pmec-4*::*mouse panx1*:: SL2::*tag rfp, Punc-122*::*gfp*] |
| AQ3776 | *unc-7(e5) bzIs17[Pmec-4::yc2.12, lin-15(+)*] ljEx973[*Pmec-4::mouse panx2*:: SL2::*tag rfp, Punc-122*::*gfp*] |
| AQ2145 (Chatzigeorgiou et al., 2010) | ljEx217[*Pegl-46*::*yc2.3*] |
| AQ2703 (Chatzigeorgiou et al., 2010) | *unc-7(e5)* ljEx217[*Pegl-46*::*yc2.3*] |
| AQ3777 | *unc-7(e5)* ljEx217[*Pegl-46*::yc2.3] ljEx956[*ser-2*prom3::*unc-7a* C173A, C191A, C377A, C394A:: SL2::*tag rfp*, *Punc-122::mcherry*] |
| AQ2232 | *lite-(ce319)* ljIs111[*Pmec-4*::ChR2] |
| AQ2214 | *mec-4(u253) lite-(ce319)* ljIs111[*Pmec-4*::ChR2] |
| AQ3336 | *lite-(ce319)* ljIs111[*Pmec-4*::ChR2] ljEx500[*Pmec-7*::*unc-7* RNAi, *Punc-122*::gfp] |
| AQ3337 | *lite-(ce319)* ljIs111[*Pmec-4*::ChR2] ljEx613[*Pmec-4*::*unc-7a* C173A, C191A, C377A, C394A:: SL2::*tagrfp*, *Punc-122*::*gfp*] |
| AX3931 (Fenk and de Bono, 2015) | dbEx651[*Psra-9::yc3.60, Punc-122::mcherry*] |
| AQ3778 | dbEx651[*Psra-9::yc3.60, Punc-122::mcherry*] ljEx793[*Psra-9::unc-7a* C173A, C191A, C377A, C394A::SL2::*tag rfp*; *Punc-122::gfp*] |
| AQ3779 | dbEx651[*Psra-9::yc3.60*; *Punc-122::mcherry*] ljEx957[*Psra-9::mec-4::mcherry, Pmyo-2::mcherry*] |
| AQ3780 | dbEx651[*Psra-9::yc3.60*; *Punc-122::mcherry*] ljEx793[*Psra-9::unc-7a* C173A, C191A, C377A, C394A::SL2::*tag rfp*; *Punc-122::gfp*] ljEx957[*Psra-9::mec-4::mcherry; Pmyo-2::mcherry*] |
| AQ3781 | dbEx651[*Psra-9::yc3.60; Punc-122::mcherry*] ljEx793[*Psra-9::unc-7a* C173A, C191A, C377A, C394A::SL2::*tag rfp*; *Punc-122::gfp*] ljEx958[*Psra-9::mec-2a::SL2-tagrfp, Pmyo-2::mcherr*y] |
| AQ3782 | dbEx651[*Psra-9::yc3.60*; *Punc-122::mcherry*] ljEx957[*Psra-9::mec-4::mcherry*; *Pmyo-2::mcherry*] ljEx963[*Psra-9::mec-2a*::SL2-*tag rfp*, *Punc-122::gfp*] |
| AQ908 (Suzuki et al., 2003) | *mec-4(u253)* bzIs17[*Pmec-4::yc2.12*; *lin-15*(+)] |
| AQ3604 | *unc-7(e5)* bzIs17 [*Pmec-4::yc2.12*; *lin-15*(+)] ljEx871[*Pmec-4::mec-4::mcherry*, *Punc-122::gfp*] |
| AQ3362 | *mec-4(u253)* bzIs17[*Pmec-4::yc2.12; lin-15*(+)] ljEx703[*Pmec-4::unc-7a* C173A, C191A, C377A, C394A, *Punc-122::gfp*] |
| AQ3356 | *mec-10(tm1552) mec-4(u253)* bzIs17[*Pmec-4::yc2.12; lin-15*(+)] ljEx703[*Pmec-4::unc-7a* C173A, C191A, C377A, C394A::SL2 *tag rfp*, *Punc-122::gfp*] |
| AQ3734 | ljEx961[*Pmec-4::unc-7::gfp*, *Pmec-4::mec-4::mcherry, rol-6(su1006)*] |
| AQ3785 | *mec-4(u253)* bzIs17 [*Pmec-4::yc2.12; lin-15*(+)] ljEx962[*Pmec-7::unc-7* RNAi, *Punc-122::gfp*] |
| AQ4895 | dbEx804[*Ptrx-1::YC3.60; Punc-122::gfp*] ljEx4895 [Ptrx-1:: *unc-7a* C173A, C191A, C377A, C394A, *Punc-122::mcherry*] |
| AX5828 (Fenk and de Bono, 2015) | dbEx804[*Ptrx-1::YC3.60; Punc-122::gfp*] |
| AQ4893 | bzIs17 [*Pmec-4::yc2.12; lin-15*(+)] ljEx1482 [*Pnmr-1::unc-7* RNAi, *Punc-122::gfp*] |
| AQ4894 | bzIs17 [*Pmec-4::yc2.12; lin-15*(+)] ljEx1483 [*Pmnr-1::unc-9* RNAi, *Punc-122::gfp*] |
